# Effect of Nb Content and Second Heat Cycle Peak Temperatures on Toughness of X80 Pipeline Steel

**DOI:** 10.3390/ma16247632

**Published:** 2023-12-13

**Authors:** Yuefeng Chen, Yaobin Yang, Xiaodong He, Qiang Chi, Lihua Qi, Weiwei Li, Xin Li

**Affiliations:** 1 National Key Laboratory of Oil and Gas Drilling and Production Transportation Equipment, Tubular Goods Research Institute CNPC, Xi’an 710077, China; 2 International Welding Technology Center, Xi’an 710077, China

**Keywords:** X80 pipeline steel, Nb, thermal simulation, HAZ, toughness

## Abstract

The microstructure evolution and variation of impact toughness in the heat-affected zone (HAZ) of X80 pipeline steel with different Nb content under different peak temperatures in the secondary thermal cycle were studied through welding thermal simulation, the Charpy impact test, EBSD analysis, SEM observation, and TEM observation in this study. The results indicate that when the peak temperatures of the second pass were lower than A_c1_, both X80 pipeline steels had high impact toughness. For secondary peak temperatures in the range of A_c1_ to A_c3_, both X80 pipeline steels had the worst impact toughness, mainly due to the formation of massive blocky M-A constituents in chain form on grain boundaries. When the secondary peak temperatures were higher than A_c3_, both X80 pipeline steels had excellent impact toughness. Smaller grain size and higher proportions of HAGBs can effectively improve the impact toughness. Meanwhile, high Nb X80 pipeline steel had higher impact absorption energy and smaller dispersion. Adding an appropriate amount of Nb to X80 pipeline steel can ensure the impact toughness of SCCGHAZ and SCGHAZ in welded joints.

## 1. Introduction

As a representative high-strength low alloy (HSLA) steel, X80 pipeline steel, with excellent strength and toughness, is widely used in the field of oil and gas transportation [1,2,3]. With the construction of long-distance oil and gas pipelines in China, the manufacturing technology of X80 pipeline steel has been intensely developed [4]. Welding is an important process in the production and laying of oil and gas pipeline steel pipes. Multi-pass welding is widely used in pipeline manufacturing and field girth welding [5,6,7,8,9]. Compared to the base material, the thermal cycle experiences a series of peak temperatures during welding that can alter the microstructure and properties in the heat-affected zone (HAZ). An important issue currently limiting the development of X80 pipeline steel is the significant difference in microstructure and mechanical performance between welded joints and base materials, with welds becoming the weakest part of X80 long-distance oil and gas pipelines [10,11,12].

According to the different peak temperatures in the secondary thermal cycle, the coarse-grain heat-affected zone (CGHAZ) can be divided into the super-critical coarse-grain heat-affected zone (SCCGHAZ), inter-critical coarse-grain heat-affected zone (ICCGHAZ), and sub-critical coarse-grain heat-affected zone (SCGHAZ) [13,14,15]. The peak temperatures in the secondary thermal cycle corresponding to the SCCGHAZ and SCGHAZ are higher than A_c3_ and lower than A_c1_, respectively. The ICCGHAZ is the pre-existing CGHAZ that was reheated to a temperature between A_c1_ and A_c3_ by a subsequent weld pass. Extensive research results have shown that the worst impact toughness in the HAZ of multi-pass welding is achieved for the ICCGHAZ, while the impact toughness of the SCCGHAZ and SCGHAZ in the HAZ is relatively high [16]. Due to the existence of martensite-austenite (M-A) islands, the impact toughness of HAZ is prone to fluctuations after the secondary thermal cycle [17,18,19]. The main reasons for the formation of the embrittlement zone are the M-A islands of the ICCGHAZ, with a chain form distributed at the grain boundary and cracking easily spread along or through the M-A [10,20,21]. Some investigations have shown that the volume fraction, size, and distribution of M-A islands can affect the low-temperature toughness of the welding joints of pipeline steel [22,23]. A higher volume fraction, larger size, and smaller spacing between M-A islands distributed on the grain boundary reduce the low-temperature toughness of the welded joints. The influence of the different secondary peak temperatures on the HAZ of the welded joint mainly comprises the change of hardness, strength, and impact toughness of the ICCGHAZ in the welded joint [24]. Results have indicated that under low heat input, the impact toughness of the ICCGHAZ decreases slightly and can be improved [25,26]. However, under large heat input, the impact toughness decreases obviously, and the impact toughness of the ICCGHAZ seriously deteriorates. At the same time, some scholars have studied the effect of Nb content on the impact toughness of the welding HAZ of HSLA steels. For example, some scholars believe that high Nb can narrow the welded CGHAZ and ensure a small grain size, which is conducive to improving the impact toughness of the CGHAZ [27]. Zhang et al. believe that the Nb element can promote the formation and coarsening of coarse bainite, expand the width of the CGHAZ, and reduce the toughness of HAZ of HSLA steel [28]. Although some studies have been carried out on the impact toughness of Nb on the HAZ of primary welding, there are few reports on the effect of different Nb content on the impact toughness of the ICCGHAZ of X80 pipeline steel.

In order to clarify the influence of different Nb content in X80 pipeline steels on the impact toughness of the HAZ of secondary thermal cycle welding, two X80 pipeline steels with different Nb content were selected as research objects in this study. The microstructure, high angle grain boundary distribution, and toughness of X80 pipeline steel with different Nb content in the HAZ of secondary thermal cycle welding were investigated using the welding thermal simulation test, which provided theoretical support for composition optimization of X80 pipeline steel with high weldability.

## 2. Experimental Procedures

### 2.1. Materials

Two X80 SMAL (longitudinal submerged arc welding) steel pipes with different Nb content were selected in this study, with dimensions of OD 1219 mm × 22 mm. Both pipes were produced by the same pipe factory, and the steel plates used were from the same steel mill in China. The chemical composition of the two X80 pipeline steels used as experimental steels is listed in Table 1. In addition to the slight difference in Nb content, there was no significant difference in the content of other alloying elements in the two X80 pipeline steels.

**Figure 1:**
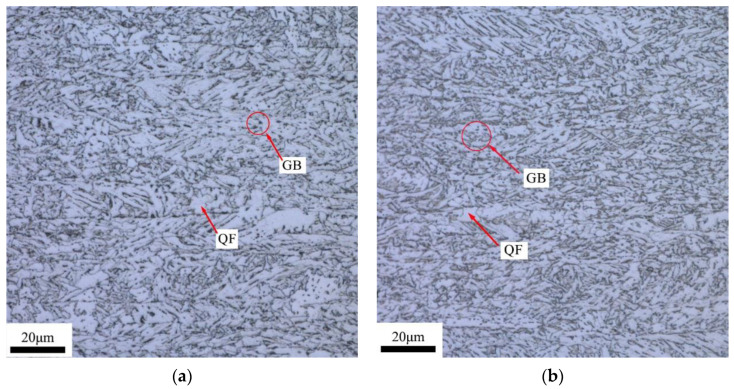
Optical micrographs of two X80 pipe steels (**a**) A and (**b**) B.

The microstructures in the center of the wall thickness of A and B pipeline steel base materials are shown in Figure 1. As depicted in Figure 1, the metallographic structures of the two pipeline steels exhibit a typical acicular ferrite morphology, consisting of granular bainite (GB) and quasi-polygonal ferrite (QF). By comparing the metallographic structures of the two pipeline steels, it was found that the content of QF in A pipeline steel was higher than that in B pipeline steel, while the content of GB was lower than that in B pipeline steel. The grain size of the two pipeline steels were measured in optical images via the intercept method according to ASTM E112 [29]. The results indicated that the grain sizes of the two pipeline steels were grade 11.5, and both steels exhibited fine grain sizes. Despite the similar grain sizes of the two pipeline steels, the grain size of B pipeline steel was smaller and more uniform than that of A pipeline steel.

**Figure 2:**
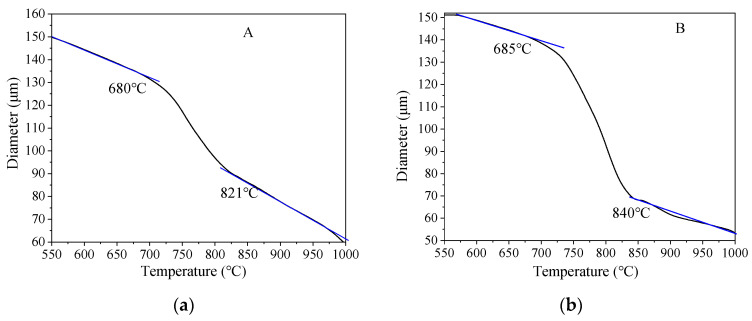
A_c1_ and A_c3_ of two samples: (**a**) A and (**b**) B.

A Gleeble-3500 thermomechanical simulator (Texas Dynamic Systems Inc., Austin, TX, USA) equipped with a thermal dilatometer was used to test the A_c1_ and A_c3_ of the two samples. Round rod specimens with dimensions of φ6 × 70 mm, taken from the center of the wall thickness along longitudinal direction of the steel pipe, were used to test A_c1_ and A_c3_. The heating rate used for A_c1_ and A_c3_ tests was 0.05 °C/s, from room temperature to 1000 °C. The variation curve of expansion of the two kinds of pipeline steel with temperature are shown in Figure 2. As the temperature increased, the crystal structure of pipeline steel changed from body-centered cube (BCC) to face-centered cube (FCC), accompanied by a sudden change in the expansion amount. The A_c1_ and A_c3_ of the two pipeline steels were determined by the change in the slope of the expansion curve. The A_c1_ and A_c3_ of sample A were 680 °C and 821 °C, respectively, while the A_c1_ and A_c3_ of sample B were 685 °C and 840 °C, respectively.

### 2.2. Welding Thermal Simulation

**Figure 3:**
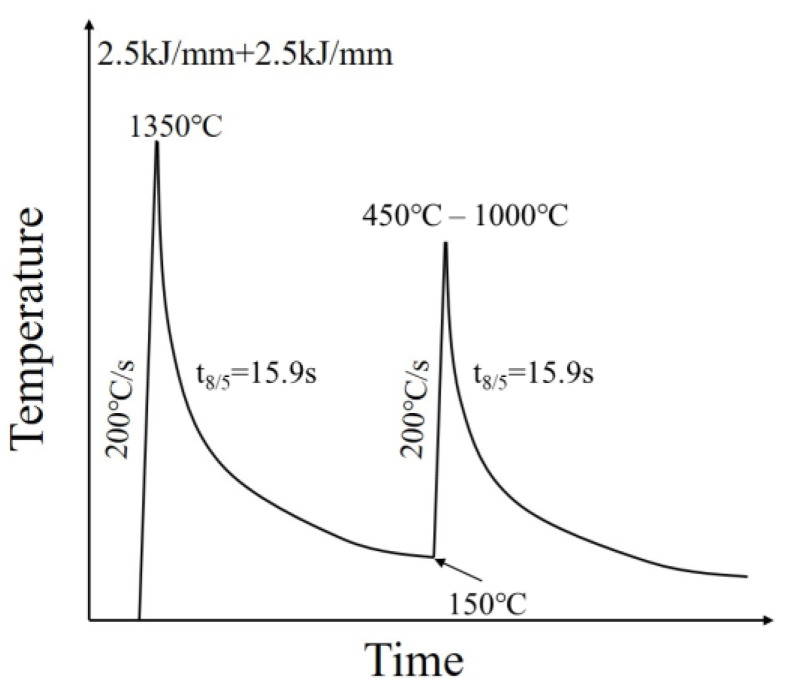
Welding thermal simulation processes diagram.

In order to simulate the changes in microstructure and impact toughness of different areas in the HAZ of X80 pipeline steel during the multi-pass welding process, samples with dimensions of 10.5 mm × 10.5 mm × 70 mm, taken from the center of the wall thickness along the longitudinal direction of the steel pipe, were used in this study. Subsequently, thermal simulation tests of the HAZ with different peak temperatures in the secondary thermal cycle were conducted on a Gleeble-3500 thermal simulator. To precisely control the temperature, K-type thermo-couples were spot-welded in the middle of the sample. Welding thermal simulation processes and parameters are shown in Figure 3. The specimens were first heated to 1350 °C at a rate of 200 °C/s and cooled to 150 °C, to simulate the CGHAZ of the first welding pass. The peak temperatures selected for the secondary thermal cycle were as follows: 450 °C, 500 °C, 550 °C, 600 °C, 650 °C, 700 °C, 725 °C, 750 °C, 775 °C, 800 °C, 825 °C, 850 °C, 900 °C, and 1000 °C, with a heat rate of 200 °C/s. The cooling time from 800 °C to 500 °C (t_8/5_) was 15.9 s for the cooling regimes of all thermal cycles, corresponding to the heat input of 2.5 kJ/mm. This heat input was simulated for the shielded metal arc welding (SMAW) process.

### 2.3. Mechanical Properties

The specimens were machined into Charpy impact specimens with sizes of 10 mm × 10 mm × 55 mm after the thermal simulation tests. The notch positions of V-type Charpy impact specimens were consistent with those of thermocouple. The impact toughness of the thermal simulated samples was tested at −10 °C.

### 2.4. Microstructure Analysis

The surfaces of the thermal simulation samples used for microstructure observation and analysis were the cross-sections where the thermocouples were located in the thermal simulation sample. Samples used for metallographic and scanning electron microscopy (SEM) observation were ground with a series of SiC papers, mechanically polished using a 1.5 μm diamond paste, and etched with 4% nitrate alcohol solution, sequentially. Electron back-scattered diffraction (EBSD) specimens were electrochemically polished in 5% perchloric acid solution after mechanical polishing. AztecCrystal Version 2.1 software was used to post-process EBSD data. Specimens with a diameter of 3 mm for the transmission electron microscopy (TEM) observation were sliced into 50 μm through abrasion on variant grit silicon carbide papers and electropolished using 90 vol.% ethanol and 10 vol.% perchloric acid. An Axiovert 405 M (Opton, Germany) metallographic microscope, TESCAN CLARA (TESCAN, Brno, Czech Republic) SEM equipped with an EBSD, and JEM-200CX (JEOL Ltd., Japan) TEM were used to characterize the microstructure and impact fracture morphology.

The samples used for microstructure and fracture analysis were numbered based on the different peak temperatures of the second pass. When the peak temperature in the secondary thermal cycle was 550 °C, samples A and B were named A-550 °C and B-550 °C. When the peak temperature in the secondary thermal cycle was 700 °C, samples A and B were named A-700 °C and B-700 °C. When the peak temperature in the secondary thermal cycle was 1000 °C, samples A and B were marked A-1000 °C and B-1000 °C.

## 3. Results and Discussion

### 3.1. Impact Toughness

**Figure 4:**
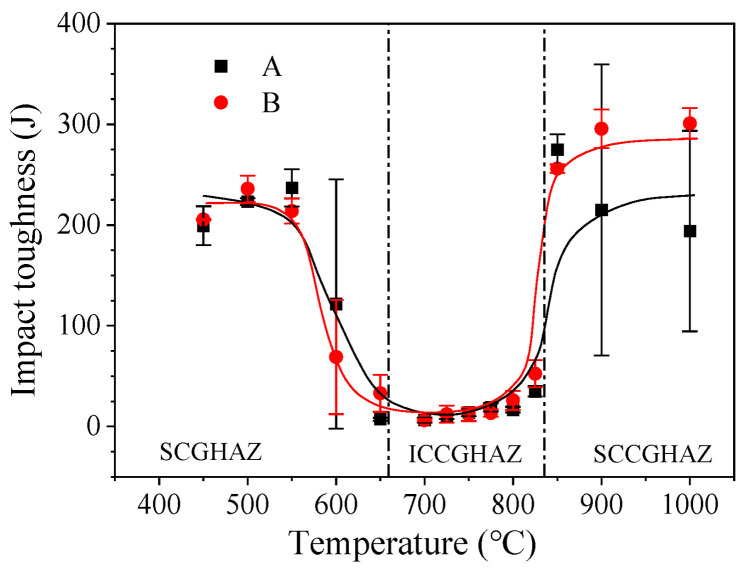
Impact toughness of two X80 steels with different peak temperatures for secondary thermal cycle.

The impact absorption energy of A and B X80 pipeline steel base material at −10 °C was 352 J and 335 J, respectively. The impact absorption energy of the two X80 pipeline steels in the weld HAZ at different peak temperatures of the secondary heat cycle is shown in Figure 4. According to the corresponding temperatures of A_c1_ and A_c3_ of the two pipeline steels in Figure 2, the secondary peak temperature corresponding to the SCGHAZ was <680 °C, the peak temperature of the secondary thermal cycle corresponding to the ICCGHAZ was 680~840 °C, and the secondary peak temperature corresponding to the SCCGHAZ was >840 °C. 

By fitting the impact absorption energy corresponding to different peak temperatures of the secondary heat cycle, the trends in the variation of the secondary peak temperature of the two pipeline steels were basically the same. With the change of secondary peak temperature, there were three platforms in the absorption energy curve, corresponding to the SCGHAZ, ICCGHAZ and SCCGHAZ, respectively. According to the change of the curve of impact absorption energy, it was obvious that the SCGHAZ and SCCGHAZ had higher impact absorption energy than the ICCGHAZ.

When the peak temperature of the second pass was below 600 °C, it corresponded to the SCGHAZ in multi-pass welded joints. The impact absorption energy values of the SCGHAZ samples corresponding to the same peak temperature of samples A and B were basically the same. For the secondary peak temperature of 450 °C, the average absorption energy values of samples A and B had no obvious difference, and both of them were about 200 J. There was a slight advantage in impact toughness of sample B for the secondary peak temperature of 500 °C, while sample A had a slight advantage for the secondary peak temperature of 550 °C. The values of average absorption energy of samples A and B for the secondary peak temperature of 500 °C were 223 J and 236 J, respectively. At a secondary peak temperature of 550 °C, the values of average absorption energy of samples A and B were 237 J and 214 J, respectively. For the thermal simulation sample with the secondary peak temperature of 600 °C, the average and dispersion absorption energy values of sample A were higher than those of sample B, but the difference in the average absorption energy values of the two samples was about 50 J. When the secondary peak temperature was 650 °C, the impact toughness values of samples A and B both significantly decreased. The average impact absorption energy value of sample A was 7 J, while the average absorption energy value of sample B was 33 J. 

When the peak temperatures of the second pass were in the range of 680–840 °C, it corresponded to the ICCGHAZ in multi-pass welded joints. A low valley appeared in the impact absorption energy curve, indicating that the toughness of the ICCGHAZ sample was the lowest among the two X80 pipeline steels. The average absorption energy values of the ICCGHAZ sample were basically the same, with minimum impact absorption energy values of 6 J. For sample A, when the secondary peak temperatures were within the range of 700–800 °C, the values for average absorption energy were less than 20 J. At reheated temperatures for sample B in the range of 700–775 °C, the average values of absorption energy were less than 20 J. The average value of impact absorption energy of sample B at the secondary peak temperature of 800 °C was 26 J. For the secondary peak temperature of 825 °C, the average absorption energy values of samples A and B were 35 J and 53 J, respectively. 

When the peak temperature of the second pass was higher than 840 °C, it corresponded to the SCCGHAZ in multi-pass welded joints. When the secondary peak temperatures were higher than 840 °C, the impact toughness of samples A and B quickly recovered to a higher level. Compared to the ICCGHAZ, the samples of the SCCGHAZ had higher impact toughness, as seen in Figure 4. However, sample B had higher impact absorption energy and smaller dispersion. The values of average impact absorption energy of sample A at the secondary peak temperature of 850 °C, 900 °C, and 1000 °C were 275 J, 215 J, and 194 J, respectively. The average values of impact absorption energy of sample B at the secondary peak temperature of 850 °C, 900 °C, and 1000 °C were 256 J, 296 J, and 301 J, respectively.

### 3.2. Microstructure Analysis

**Figure 5:**
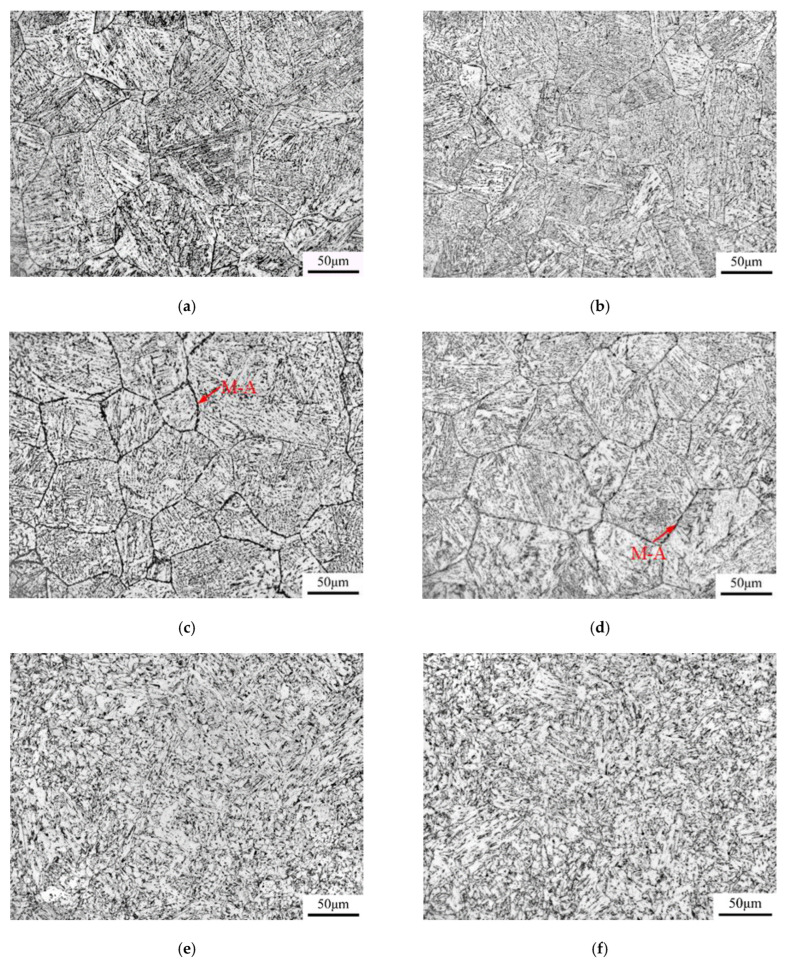
Optical micrographs of thermal simulation samples: (**a**) A-550 °C, (**b**) B-550 °C, (**c**) A-700 °C, (**d**) B-700 °C, (**e**) A-1000 °C, and (**f**) B-1000 °C.

**Figure 6:**
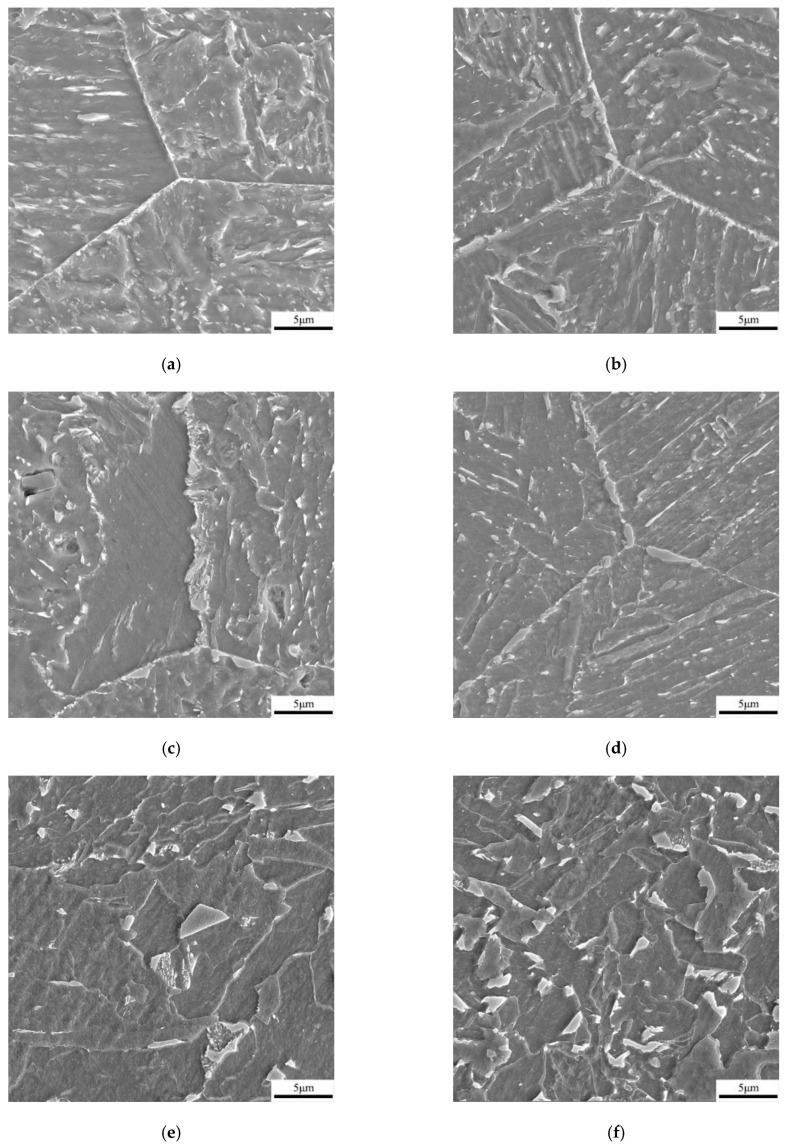
SEM micrographs of thermal simulation samples: (**a**) A-550 °C, (**b**) B-550 °C, (**c**) A-700 °C, (**d**) B-700 °C, (**e**) A-1000 °C, and (**f**) B-1000 °C.

Optical and SEM micrographs of the thermal simulated samples with different peak temperatures of the secondary cycle are presented in Figure 5 and Figure 6, respectively. When the secondary peak temperature was lower than A_c1_, both A-550 °C and B-550 °C samples maintained the characteristics of the primary CGHAZ, and the grain size was relatively coarse. The microstructures of the two thermal simulation samples were mainly dominated by GB, with a small number of M-A components distributed within the grains. By statistically measuring the grain size in ten metallographic micrographs, the average size of grains in specimens of A-550 °C and B-550 °C was 74.9 μm and 72.6 μm, respectively.

At a reheated temperature of the secondary thermal cycle between A_cl_ and A_c3_, the coarse grain microstructure formed during the first thermal cycle could not completely undergo austenite phase transformation, and only a portion of the microstructure could undergo austenite phase transformation. In the samples of A-700 °C and B-700 °C, the M-A component was generated in a blocky form at the original austenite grain boundaries, with a large number of blocky M-A components in necklace-type form. According to the statistics of grain size in specimens of A-700 °C and B-700 °C, the average sizes of grain were 69.4 μm and 68.2 μm, respectively. Through the SEM analysis of the samples with the secondary peak temperatures of 550 °C and 700 °C, it was found that the width of grain boundary increased obviously with the increase of the second pass peak temperature. This was mainly because when the secondary temperature was 700 °C, a large number of massive M-A components were formed on the grain boundaries, which made the grain boundary width larger.

For the samples of secondary peak temperature higher than A_c3_, the CGHAZ microstructure that occurred in the first pass was completely austenitized, but the peak temperature of the second pass had not yet reached the temperature of rapid grain growth and coarsening, resulting in smaller austenite grains that form polygonal ferrite (PF) and QF during the cooling process. At the same time, a small amount of fine and regularly shaped M-A in the second pass was also formed at the grain boundaries. The grain size of samples with a secondary peak temperature of 1000 °C were measured by EBSD, with average sizes of grain of 26.5 μm and 24.7 μm for samples A-1000 °C and B-1000 °C, respectively.

**Figure 7:**
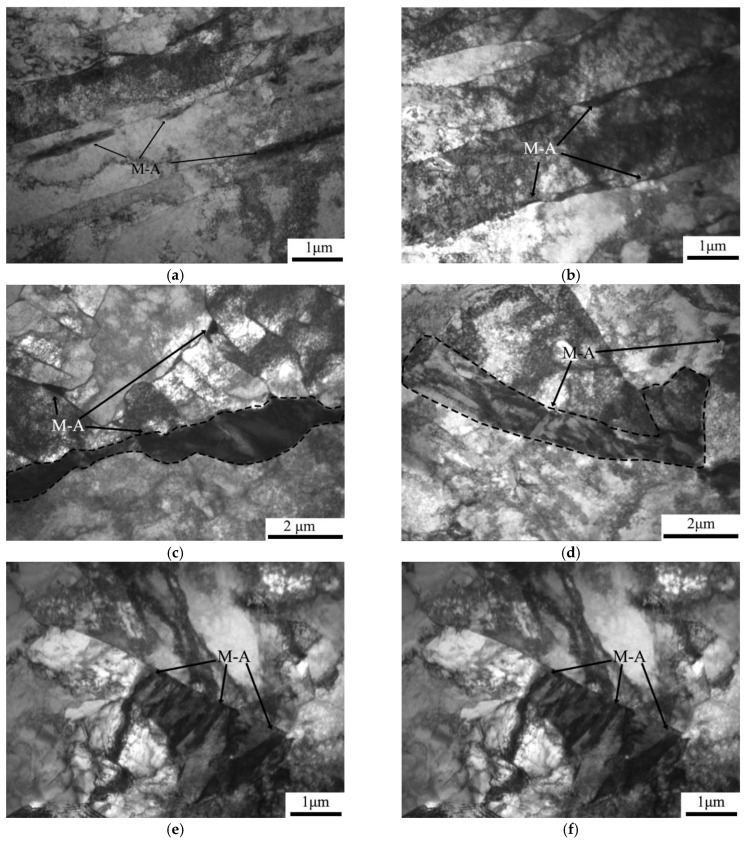
TEM micrographs of thermal simulation samples: (**a**) A-550 °C, (**b**) B-550 °C, (**c**) A-700 °C, (**d**) B-700 °C, (**e**) A-1000 °C, and (**f**) B-1000 °C.

The TEM characterization of the microstructures of thermal simulation samples is depicted in Figure 7. In the TEM morphology of all thermal simulated samples, layer-by-layer structures appeared in the form of dark-light-dark. This kind of structure was also found in the reference studied by Li et al. [20,30], which was considered to have typical M-A constituents. When the secondary peak temperature was 550 °C, the microstructure of A-550 °C and B-550 °C samples was dominated by parallel lath ferrite with high dislocation density and thin film M-A components distributed between the paralleled lath ferrite. These nearly parallel laths were considered to have almost the same crystallographic orientation. According to the statistics of the width of ferrite laths in the samples, the width of ferrite laths in the A-550 °C specimen was about 2.2 μm, while the width of ferrite laths in the B-550 °C specimen was about 2.7 μm. For the secondary temperature of 700 °C, a large number of blocky M-A island components appeared on the grain boundaries of A-700 °C and B-700 °C samples, which were in chain form. At a reheated temperature of 1000 °C, the ferrite matrix was in the form of lath or quasi-polygon, and the dislocation density inside the ferrite laths was significantly reduced. M-A components were distributed between ferrite laths in the form of film or distributed at the boundary of QF in the form of particles.

### 3.3. Fracture Morphology Analysis

**Figure 8:**
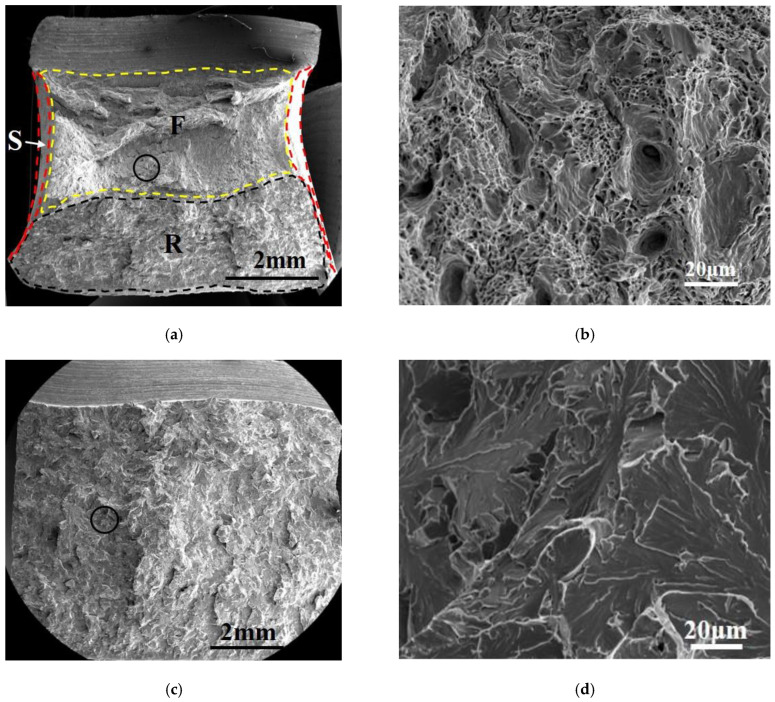

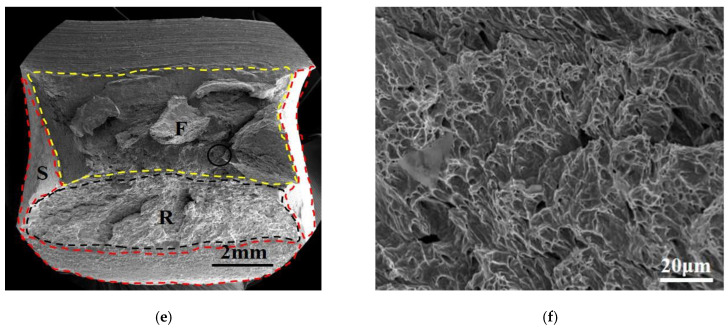
Macroscopic and microscopic fracture morphology of thermal simulation samples for specimens A: (**a**,**b**) A-550 °C, (**c**,**d**) A-700 °C, and (**e**,**f**) A-1000 °C.

Macroscopic and microscopic fracture morphology of thermal simulation samples for pipeline steel A is presented in Figure 8. The macroscopic fracture morphology of samples is shown on the left, which is divided into fiber zone (F), radiation zone (R), and shear lip zone (S). The microscopic morphology of the marked circle area in the macroscopic fracture morphology is shown on the right. For the secondary peak temperature of 550 °C, the size and depth of the dimples in the fracture surface of the sample were larger, and there was a certain proportion of cleavage zones. When the secondary peak temperature was 700 °C, the fracture surface was nearly in the radiation zone. The entire fracture surface was almost entirely cleavage fracture, with a river-like pattern and no dimples present. For the reheated peak temperature of 1000 °C, the fiber zone was mainly composed of dimples of varying sizes. Compared with the sample at A-550 °C, the number of dimples decreased, and they were shallower, while the number of cracks increased. The area of the cleavage zone, size of the cleavage step, and number of dimples at the tearing edge were significantly decreased.

**Figure 9:**
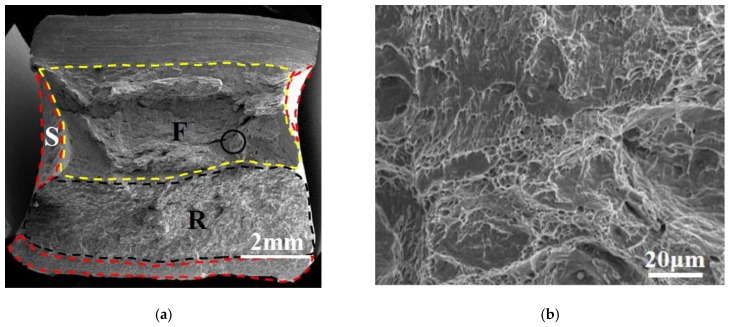

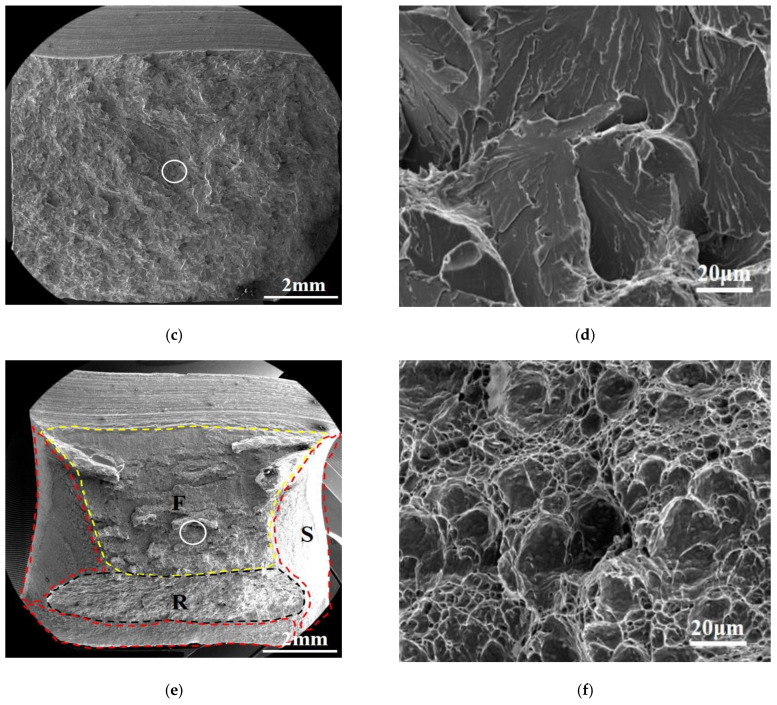
Macroscopic and microscopic fracture morphology of thermal simulation samples for specimens A: (**a**,**b**) B-550 °C, (**c**,**d**) B-700 °C, and (**e**,**f**) B-1000 °C.

The macroscopic and microscopic fracture morphology of thermal simulation samples for pipeline steel B is presented in Figure 9. The microscopic morphology of the marked circle area in the macroscopic fracture morphology is shown on the right. Compared with the A-550 °C sample, the depth of dimples in the fracture surface of the B-550 °C sample was shallower, the size was smaller, and the cleavage zone area was slightly larger. When the secondary peak temperature was 700 °C, similar to the A-700 °C sample, the fracture surface was nearly in the radiation zone. The entire fracture surface was almost entirely of cleavage character, with a river-like pattern, and no dimples were present. For the secondary peak temperature of 1000 °C, the shear lip zone ratio was the highest among all thermal simulation samples, indicating that this sample had experienced obvious plastic deformation under the impact load. Therefore, the sample B-1000 °C had high plastic toughness and excellent impact toughness. In the microscopic fracture morphology of sample B-1000 °C, there was a large number of deep and large-sized dimples in the fracture surface, and the cleavage zone area was relatively small.

### 3.4. Discussion

**Figure 10:**
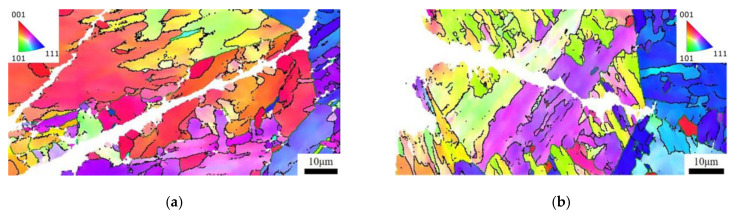

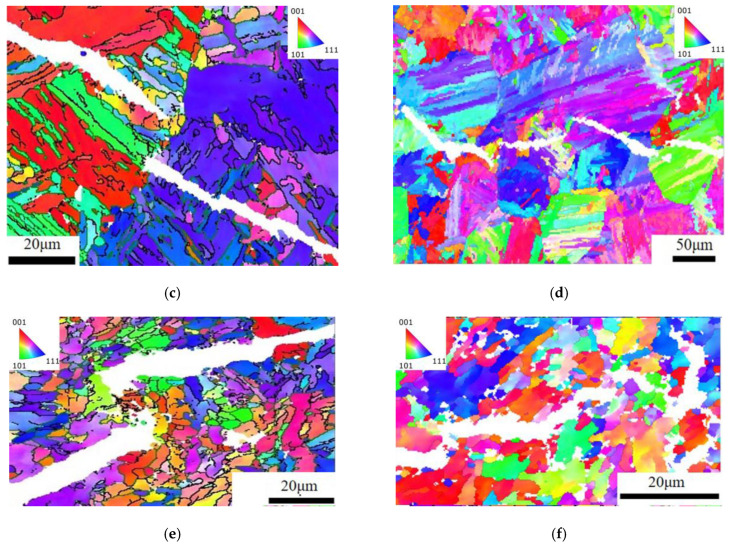
Propagation path of secondary cracks of simulation samples: (**a**) A-550 °C, (**b**) B-550 °C, (**c**) A-700 °C, (**d**) B-700 °C, (**e**) A-1000 °C, and (**f**) B-1000 °C.

The EBSD maps of the secondary crack close to the main crack of the Charpy impact specimens with different reheated peak temperatures are presented in Figure 10. When the peak temperatures of the secondary cycle were 550 °C and 700 °C, the cracks propagated directly in the grain without deflection and turned when encountering grain boundaries. When the peak temperature of the secondary cycle was 1000 °C, the crack did not extend in a straight line but bended going forward.

**Figure 11:**
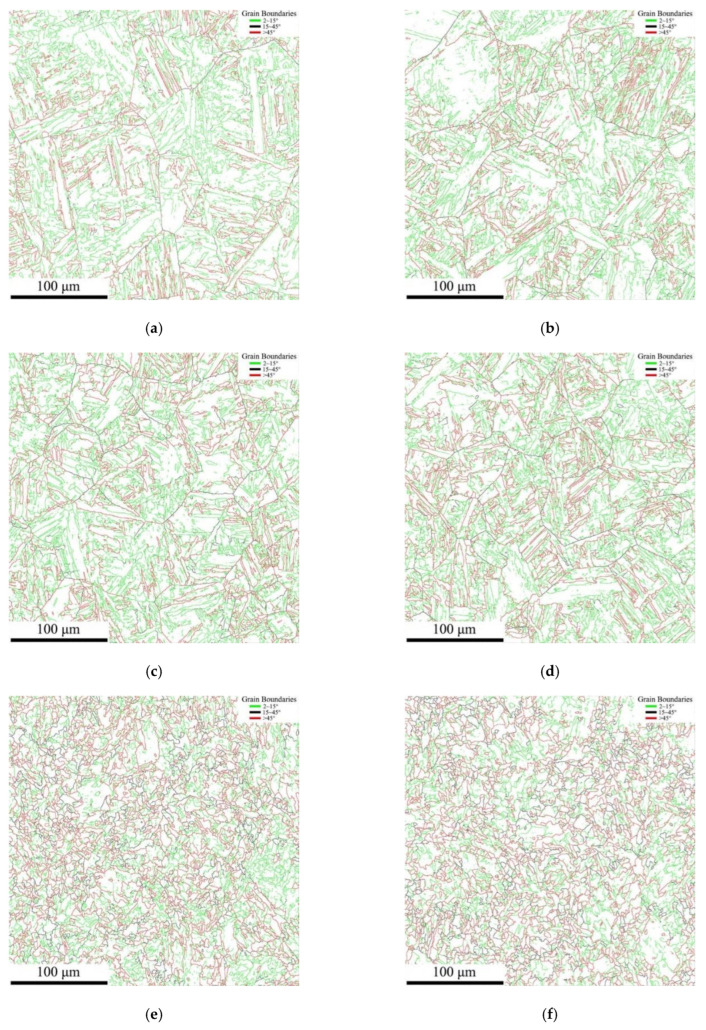
Grain boundary distribution of thermal simulation specimens: (**a**) A-550 °C, (**b**) B-550 °C, (**c**) A-700 °C, (**d**) B-700 °C, (**e**) A-1000 °C, and (**f**) B-1000 °C.

**Figure 12:**
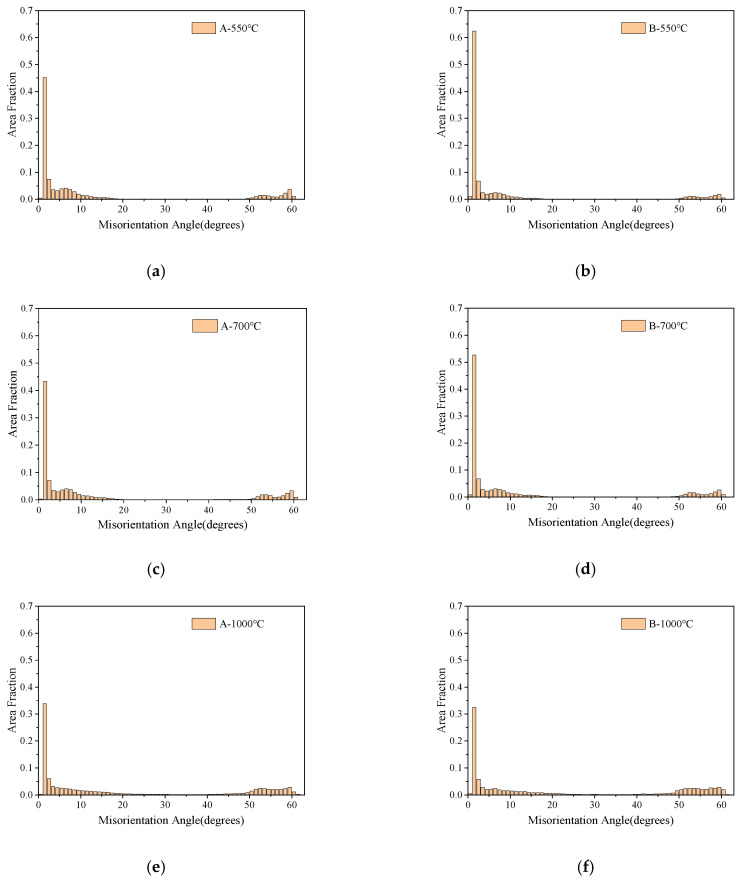
Grain orientation angles of thermal simulation specimens: (**a**) A-550 °C, (**b**) B-550 °C, (**c**) A-700 °C, (**d**) B-700 °C, (**e**) A-1000 °C, and (**f**) B-1000 °C.

The grain boundary maps and misorientation angle maps of thermal simulation samples at different peak temperatures of the second thermal cycle are shown in Figure 11 and Figure 12, respectively. As presented in Figure 11, the misorientation angle within the range of 2~15° is regarded as low-angle grain boundaries (LAGBs), marked in green lines, the black line indicates the medium-angle grain boundaries (MAGBs) with the misorientation angle between 15° and 45°, while a misorientation angle higher than 45° is characterized as high-angle grain boundaries (HAGBs), marked in red lines. It can be seen from Figure 11 and Figure 12 that only a small amount of MAGBs existed in all the thermal simulation samples. As illustrated in Figure 11a,b, there were HAGBs between the lath of the bainite structure and the grain boundaries in the A-550 °C and B-550 °C samples, while the LAGBs were only distributed inside the laths. As presented in Figure 11e,f, for the A-1000 °C and B-1000 °C samples, the grain boundaries of PF and QF were HAGBs, and the interior of the QF was LAGBs. 

The statistical results of grain boundary lengths classified from different misorientation angles are shown in Table 2. According to the results of Table 2 and Figure 12, quantitative statistical analysis was conducted on LAGBs, MAGBs, and HAGBs, and the statistical results are displayed in Table 3. There was no significant difference in length and proportion for HAGBs, MAGBs, and LAGBs in samples A and B for secondary peak temperatures of 500 °C and 700 °C, which was mainly related to the very close grain size of the thermal simulated samples at the two peak temperatures. However, the length and proportion of MAGBs and HAGBs in the thermal simulation sample with a secondary peak temperature of 1000 °C increased significantly, which corresponded to the refinement of grain size in the samples. 

Although the fractions of HAGBs, MAGBs, and LAGBs in the thermal simulation samples for secondary peak temperatures of 550 °C and 700 °C were basically the same, the corresponding impact toughness was quite different. According to the metallographic, SEM and TEM morphology analysis, a large number of M-A components were distributed on the grain boundaries of A-700 °C and B-700 °C samples compared with A-550 °C and B-550 °C samples. The results of Li et al. revealed that when the M-A component length is greater than 2 μm and 1 μm in thickness, this kind of M-A constituent can promote nucleation of the brittle fracture either through cracking or debonding from the matrix. For the secondary peak temperature of 1000 °C, the grains in A-1000 °C and B-1000 °C samples were obviously refined. Therefore, the length and ratio of MAGBs and HAGBs in the B-1000 °C sample were the highest among the thermal simulation samples, corresponding to the highest impact absorption energy and lower dispersion degree of the impact absorption energy values. The increase in the proportions of HAGBs helped to prevent the propagation of cracks, so that more energy was consumed during the propagation process, thus improving the impact toughness of the material [27,28].

## 4. Conclusions

The effect of different peak temperatures in the second pass on the impact toughness of X80 pipeline steel with different Nb contents, microstructure, and fracture morphology of the thermal simulation samples was investigated in this study. The main conclusions are derived as follows.

1. The secondary peak temperatures corresponding to the toughness valley of the two X80 pipeline steels were located at A_c1_ to A_c3_. For the peak temperature of the second pass lower than A_c1_, both X80 pipeline steels had high impact toughness, and the impact absorption energy was similar for both steels. When the secondary peak temperatures were higher than A_c3_, the X80 pipeline steel with higher Nb content had excellent impact toughness, lower impact dispersion, and higher impact absorbed energy than the X80 pipeline steel with lower Nb content.
2. When M-A components were distributed at the ferrite lath interface in a film-like form or in smaller sizes, they had less impact on the impact toughness of the HAZ of X80 pipeline steel. When chain-like distributed massive M-A constituents appeared on the grain boundaries, the impact toughness of the HAZ of X80 pipeline steel was seriously deteriorated.
3. Adding an appropriate amount of Nb to X80 pipeline steel can improve the impact toughness of the SCCGHAZ and reduce the dispersion degree of impact absorption energy values.

## Figures and Tables

**Table 1:** Chemical composition of two pipeline steels (wt%).

Sample	C	Si	Mn	Cr	Mo	Ni	Nb	V	Ti	Cu	Al
A	0.049	0.15	1.73	0.25	0.093	0.16	0.058	0.0043	0.011	0.022	0.027
B	0.049	0.15	1.72	0.26	0.090	0.16	0.084	0.0045	0.011	0.026	0.023

**Table 2:** Grain boundary length of thermal simulation specimens for different misorientation angles (mm).

Sample	A-550 °C	A-700 °C	A-1000 °C	B-550 °C	B-700 °C	B-1000 °C
2~15°	26.83	27.15	20.74	26.84	27.29	17.17
15~45°	2.29	2.76	7.28	2.15	2.99	8.44
>45°	13.68	13.83	17.65	13.90	13.54	19.57

**Table 3:** Ratio of grain boundary for different misorientation angles (%).

Sample	A-550 °C	A-700 °C	A-1000 °C	B-550 °C	B-700 °C	B-1000 °C
2~15°	62.69	62.08	45.42	62.58	62.28	38.00
15~45°	5.36	6.30	15.93	5.00	6.83	18.69
>45°	31.96	31.63	38.65	32.42	30.89	43.31

## Data Availability

Data is contained within the article.
